# Editorial: Biocompatible and Biodegradable Polymer Materials

**DOI:** 10.3390/polym18101245

**Published:** 2026-05-20

**Authors:** Lorenzo A. Picos-Corrales, Grégorio Crini, Elizabeth Carvajal-Millan

**Affiliations:** 1 Facultad de Ingeniería Culiacán, Universidad Autónoma de Sinaloa, Ciudad Universitaria, Culiacan 80013, Sinaloa, Mexico; 2 Laboratoire Chrono-Environnement, Université Marie et Louis Pasteur, 16 Route de Gray, 25000 Besançon, France; 3 Centro de Investigación en Alimentación y Desarrollo, A.C. (CIAD, AC), Carretera Gustavo Enrique Astiazarán Rosas No. 46, Hermosillo 83304, Sonora, Mexico

Biocompatible and biodegradable polymer materials offer essential properties in systems designed to protect human health, preserve food products, and improve water treatment, among other uses. In this regard, developing innovative solutions is crucial, such as for achieving controlled drug delivery with minimal side effects [1,2,3]; extending food shelf life by regulating moisture loss, oxygen exposure, and microbial growth [4,5,6]; and effectively removing emerging contaminants from water [7,8,9]. Hence, in many cases, researchers are developing new methods and biocompatible and biodegradable polymers to overcome the inherent drawbacks of traditional materials and systems, while retaining or enhancing their key properties for high effectiveness, in line with the intended application. The Special Issue titled “Biocompatible and Biodegradable Polymer Materials” brings together original research articles and reviews that present recent research on the use of these materials. The contributions are addressed below in relation to the potential application and the type of article. This Editorial includes reports on the synergistic effect between polyaluminum chloride and chitosan in the flocculation process; the bactericidal effects of chitosan, reducing chlorine demand in water-treatment plants; the use of chitosan foams with embedded PS–FMBO for hazardous waste management; the development of LL37–peptide-loaded chitosan nanoparticles as an antimicrobial sustained-release system; the use of polydopamine-coated strontium-doped hydroxyapatite to regulate cell biological activity; the potential of polyethylenimine carriers for drug and gene delivery; the roles of scaffold materials and coatings in engineering cell microenvironments; and the potential of sodium alginate for food packaging applications.

De-Paz-Arroyo et al. studied the synergistic effect between polyaluminum chloride (PAC) and chitosan in the flocculation process for river water treatment [10]. PAC counteracted the negative surface charge of colloids, while chitosan promoted interparticle bridging, respectively. The flocculant mixture overcame the limitation of chitosan, which typically requires specific optimal dosages. In addition, the mixture required a significantly lower dosage (2.75 mg L^−1^) than inorganic polyaluminum chloride (5 mg L^−1^), yielded the best removal of a model microplastic, and produced larger and more compact flocs than the individual flocculants. Thus, the overall flocculation process was improved. Therefore, the results may be valuable for potabilization plants aiming to incorporate eco-friendly flocculants into their processes.

The bactericidal effects of chitosan were explored by Molina-Pinna et al. with the purpose of reducing chlorine requirements in a water-treatment plant [11]. Chitosan dosed in an acid solution improved the bactericidal effect, enabling the disinfection point to be reached with a lower concentration of sodium hypochlorite, compared to using this traditional disinfectant alone. Chitosan-treated samples tested negative for coliforms, whereas control samples treated solely with sodium hypochlorite remained positive. In addition, the results and cost of substances appear to justify the investment in chitosan.

Vujić et al. investigated the regeneration, reuse, and stabilization of Fe–Mn polymer nanocomposites (PS–FMBOs) for arsenic removal from water [12]. Subsequently, chitosan foams with embedded PS–FMBOs were prepared, and leaching tests confirmed low leached concentrations of As, Fe, and Mn, reducing the risk of their release into the environment. Additionally, the porous structure of chitosan foams facilitates efficient sound absorption, as evidenced by the sound absorption test. Therefore, PS–FMBOs and chitosan-based foams may be sustainable materials for hazardous waste management and eco-friendly construction applications.

LL37–peptide-loaded chitosan nanoparticles were developed by Ergün et al. [13]. The ionic gelation method allowed the preparation of spherical nanoparticles (particle size close to 211 nm and zeta potential around +51 mV), reaching an encapsulation efficiency of 80%. The formulation exhibited a release profile consistent with a first-order kinetic model, no cytotoxicity in keratinocyte cells, and significant antibacterial activity against *E. coli* and *S. aureus*. The findings revealed the potential of these LL37–chitosan nanoparticles as an antimicrobial sustained-release system.

Strontium-doped hydroxyapatite nanowires (SrHAWs) were synthesized by Li et al. using a hydrothermal method and subsequently coated with polydopamine (PDA) to improve their functional properties [14]. Specifically, a uniform PDA layer 10 nm thick was deposited on the nanowire surfaces. The PDA coating positively influenced cell proliferation and differentiation in three-dimensional composite spheroids. This system promotes enhanced cell–cell and cell–material interactions, which in turn modulate cellular activity and differentiation.

In their review article, Ismail and Chou summarized the potential of polyethylenimine carriers for drug and gene delivery [15]. Methods for obtaining nanoparticles, coatings, nanofibers, hydrogels, and films are addressed. The structure–property relationships of carriers are related to molecular weight, branching degree, and surface modification. Also, challenges in the clinical translation of these materials were identified, including cytotoxicity, non-degradability, and serum instability.

The survey by Ramírez-González et al. presents the role of scaffold materials and coatings in engineering cell microenvironments, advancing organ-on-a-chip systems [16]. For instance, molecules used to resemble the extracellular matrix are studied. In addition, coating techniques, including self-assembled monolayers, dip coating, spin coating, microcontact printing, and 3D bioprinting, are discussed. In this case, improving biocompatibility, scalability, and reproducibility are challenges listed in the text.

The review article by Schenk et al. examines the potential of sodium alginate for food packaging applications using literature on pure sodium alginate films plasticized and ionically crosslinked, avoiding modifiers or nanofillers [17]. The characteristics of the system, such as oxygen and fat barriers, water vapor permeability, and brittleness, are revised. In this topic, drying temperature, mixing speed, molecular weight, and protein incorporation are factors that influence film performance. The review also highlights existing limitations, particularly in packaging foods with high salt content. Finally, turning lab-optimized formulations into films that maintain their properties on an industrial scale is projected as the most significant research gap.

The contributions to the Special Issue collectively advance the understanding of polymers used as adsorbents of contaminants, as carriers of active compounds, and as protective barriers in food products. Notably, polymers containing amino groups (e.g., chitosan, polydopamine, and polyethylenimine) have been the most investigated, with chitosan-based materials being the most frequently used. As is well known, amino groups in a polymer backbone are highly effective in promoting host–guest interactions. In particular, chitosan is a versatile polysaccharide that exhibits biocompatibility, biodegradability, and antimicrobial effects. This polysaccharide contains –OH and –NH_2_ groups, which engage in chemical interactions with small molecules, ions, macromolecules, cell membranes, and metal surfaces through electrostatic interactions, hydrogen bonding, hydrophobic interactions, and chelation [18].

In conclusion, challenges persist in the previously discussed topics. The findings from the articles included in the Special Issue “Biocompatible and Biodegradable Polymer Materials” may be valuable for the development of the proposed applications, in certain cases, not only at the laboratory level but also at the industrial scale.

## Data Availability

Not applicable.
